# 

**Published:** 1916-08-05

**Authors:** 


### CONTENTS.

| | PAGE |
|---|---|
| **The Catholicity of Medicine** ... ... ... ... | 419 |
| **The Leeds Infirmary and a Justice of Assize** | 420 |
| **Hospital and Institutional News** ... ... ... | 421 |
| Lord Knutsford's Return to Health—Women Medical Students and Edinburgh University—A Home for Disabled Officers—A Reconstructed Medical Service—Resignation of Dr. Armstrong Jones—Wittenberg Again—An English Monastery as a Hospital—The Problem of Beds at Manchester Royal Infirmary—Mr. E. W. Morris on the Effect of the Insurance Act—An Abortive Scheme—The *Modern Hospital*—Hospital Superintendent gets £3,000 Damages—The Supply of Greens to V.A.D. Hospitals—Poor-Law Centralisation and the Irish Priests—Another "Medical Index"—Memorial to a Secretary Killed in Action—The Treatment of Measles in London—The Value of a Ladies' Auxiliary Committee—The R.A.M.C. Depot Magazine—The Contents of Your Hospital Gazette—A Dublin Charity and the Late Rebellion—An Asylum Farm—An Irish Memorial to Edith Cavell—Manor Hospital as a War Hospital—More Women Workers and Nurses Wanted—A Legacy to a Board of Guardians—The Cocaine Proclamation—Decorations for Doctors from Wittenberg Camp—This Week's Drug Market. | |
| **Surgical Operations Without Shock** ... ... | 427 |
| **How the Law Regards the Loss of One Eye...** | 428 |
| **Human Temperaments** ... ... ... ... | 429 |
| VIII.—The Practical Man. | |
| **The Institutional Library** ... ... ... ... | 431 |
| A Helpful Book: Its Story and Results. | |
| **The Cocaine Habit** ... ... ... ... ... | 432 |
| Government Action at Last. | |
| **The Matrons' and Sisters' Department** ... | 433 |
| Commandant and Matron. | |
| Round the Hospitals. | |
| Non-Aseptic Nurse Perambulators—The King George Hospital—Disgraceful Sleeping Quarters—Staff Breaking Down—Hot-Weather Grumblers—Roughness and Discourtesy to Patients. | |
| **Nurses' Registration, 1916** ... ... ... ... | 435 |
| The Bill Approved by the College Council. | |
| **From Across the Seas** ... ... ... ... | 436 |
| **The Editor's Letter-Box** ... ... ... ... | 437 |
| The London Hospital: An Immediate Need—The Sale of Medicated Wines—The Control of Lay Women in Hospitals—Well Done, Nursing Staff! Next Please—The Training for Masseuses—More V.A.D.s of all Ranks Wanted. | |
| **Literary Intelligence** ... ... ... ... ... | 438 |
| **The Book Market** ... ... ... ... ... | 438 |
| **Hospital and Institutional Results in 1915** ... | 439 |
| **Passing Events and Topics** ... ... | 440 |
| **Matrons' and Sisters' Appointments** ... ... | vi |
| **More Nurses Required for Military Hospitals** | vi |
| **Institutional Needs** ... ... ... ... ... | vi |
| **Gifts to Hospitals** ... ... ... ... ... | vi |
| **The Institutional Worker** ... ... (Suppl.) | 1 |

